# Defining the role of natural killer cells in acute myeloid leukemia through the lens of single-cell omics

**DOI:** 10.3389/fimmu.2026.1734327

**Published:** 2026-01-30

**Authors:** Chen Liang, Meichi Yue, Kehui Zhang, Sining Zhou, Xiaojing Xu, Shiwei Wang, Shiping Liu

**Affiliations:** 1 Key Laboratory of Resources Biology and Biotechnology in Western China, Ministry of Education, Provincial Key Laboratory of Biotechnology of Shaanxi Province, The College of Life Sciences, Northwest University, Xi’an, China; 2 State Key Laboratory of Genome and Multi-omics Technologies, BGI Research, Hangzhou, China

**Keywords:** acute myeloid leukemia, bone marrow microenvironment, cell immunotherapy, immune evasion, natural killer cells, single-cell omics

## Abstract

This review explores the alterations in natural killer (NK) cell function in acute myeloid leukemia (AML) and their implications for disease progression and therapeutic outcomes. As key effectors of innate immunity, NK cells are critical in recognizing and eliminating malignant cells. In AML, however, NK cells frequently exhibit numerical and functional deficiencies, resulting in compromised immunosurveillance that facilitates tumor immune escape and disease advancement. We systematically examine the application of single-cell omics technologies in AML research to elucidate the omics profiles and phenotypic distribution of NK cells within the leukemic microenvironment, characterizing their dysfunctional state by upregulated inhibitory receptors, downregulated activating signals, an altered cytokine milieu, and complex cellular crosstalk within the bone marrow (BM) niche. Furthermore, this article correlates functional dynamics of NK cells with conventional and emerging treatments, including CAR-NK immunotherapy, underscoring their potential role in disease monitoring and prognostic stratification. We also discuss promising NK cell-based immunotherapeutic strategies for AML, emphasizing the potential of modulating or engineering NK cells to enhance antitumor immunity. A deeper understanding of NK cell biology and regulatory mechanisms in AML is essential for developing novel immunotherapies and improving patient prognosis.

## 1 Introduction

Acute myeloid leukemia is a highly heterogeneous malignant clonal disorder originating from hematopoietic stem and progenitor cells. It is characterized by the aberrant proliferation of immature myeloid cells within the BM, leading to suppression of normal hematopoiesis (1–3). AML pathogenesis involves mutations in key genes such as *FLT3*, *NPM1*, *DNMT3A*, and *TET2*, alongside dysregulated epigenetic modulation, aberrant signaling pathways, and an immunosuppressive microenvironment (4). Accounting for 70%–80% of adult acute leukemias, AML demonstrates higher incidence, more rapid progression, and poorer prognosis in older adults, with a one-year mortality rate exceeding 70% in patients over 65 (5, 6). Although targeted therapies (e.g., *FLT3* or *IDH* inhibitors) and immunotherapies have recently advanced, and various immunotherapies are under active investigation, their clinical efficacy remains limited outside of allogeneic stem cell transplantation (7).High relapse rates remain a major clinical challenge, underscoring the need for more effective and personalized treatment strategies (8, 9).

Within the AML landscape, NK cells are central players in innate immunity and serve a critical role in antitumor responses (10). Unlike T cells, NK cells do not require antigen presentation and can rapidly identify and eliminate tumor cells that lack MHC-I expression or express stress-induced ligands within minutes. This rapid response capability offers a distinct advantage in fast-progressing malignancies like AML (11). NK cells induce apoptosis in target cells through perforin and granzyme release and engage activating receptors—including NKG2D, NKp30, and NKp46—to detect abnormal cells. Furthermore, CD16 (FcγRIII) facilitates antibody-dependent cellular cytotoxicity (ADCC), bridging innate and adaptive immunity (12). Critically, emerging evidence suggests NK cells hold significant therapeutic potential to induce durable remission in AML. As allogeneic products, they are not constrained by antigen specificity and are associated with a markedly lower risk of inducing graft-versus-host disease (GVHD) compared to T cells, positioning them as a safer and more readily available “off-the-shelf” cellular therapy (13).

However, in AML patients, NK cells often exhibit increased expression of inhibitory receptors and impaired cytotoxicity (14). The leukemic microenvironment further disrupts NK cell function through metabolic alterations—driven by factors such as TGF-β, PGE2, and CXCR4—resulting in mitochondrial dysfunction, aberrant glycolysis, and reduced oxidative phosphorylation (OXPHOS) (15). Collectively, these mechanisms lead to significant impairments in NK cell effector functions and metabolic fitness, rendering them susceptible to immune evasion.

The precise mechanisms driving this functional exhaustion in NK cells, however, remain comparatively less understood than the well-characterized exhaustion pathways in T cells within AML (16). This gap in knowledge is, in part, attributable to the inherent limitations of conventional bulk analytical approaches. Techniques such as bulk RNA-seq and pooled flow cytometry treat heterogeneous populations as a whole, yielding only averaged measurements that mask the cellular heterogeneity and dynamic states within the NK cell compartment, thereby obscuring the very information needed to decipher exhaustion mechanisms. Consequently, single-cell and spatial multi-omics technologies have become pivotal in overcoming this research bottleneck. Their high-resolution capacity to track cellular dynamics, identify rare populations, and preserve critical spatial information is essential for mapping the precise molecular circuits of NK cell dysfunction (17–19). This confluence of high therapeutic promise and unresolved—but now addressable—biology underscores why NK cells occupy a pivotal position in AML research. Elucidating their dysfunction through these advanced tools is not only essential for understanding immune evasion but also represents a critical and feasible frontier for developing safer, more effective immunotherapies.

While previous reviews have systematically examined NK cell dysfunction in AML from conventional immunological and clinical perspectives, a key knowledge gap persists regarding the heterogeneity, dynamic trajectories, and spatial drivers of NK cell exhaustion within the leukemic microenvironment (20, 21). This review specifically addresses this gap by focusing on the application of single-cell and spatial multi-omics technologies to deconvolute the AML niche. We detail how these high-resolution approaches move beyond bulk analyses to map the spectrum of NK cell states and their spatial interactions with leukemic cells.

These technologies provide unique insights by enabling: 1) the identification of novel NK cell subsets and exhaustion signatures, 2) the reconstruction of dysfunction trajectories, and 3) the spatial mapping of immunosuppressive niches. These insights directly guide future research and clinical practice by facilitating the discovery of predictive biomarkers, informing the rational design of NK cell-based therapies (e.g., CAR-NK), and providing a framework for evaluating treatment efficacy at a single-cell resolution. Integrating discussions on multimodal data and artificial intelligence (AI), this review aims to bridge molecular observation with therapeutic innovation, charting a path toward precision NK cell immunotherapy in AML.

## 2 Current applications and future challenges of single-cell omics in AML-NK cell research

### 2.1 Key advantages of single-cell omics in AML NK cell profiling

Conventional bulk sequencing techniques face fundamental limitations in AML research, as they cannot uncover the heterogeneity of gene expression within leukemia cell populations or delineate the evolutionary trajectories and resistance mechanisms of malignant subclones across and within patients. In contrast, single-cell omics technologies has provided revolutionary tools for dissecting the heterogeneity, functional states, and interactions of NK cells within the complex microenvironment of AML at high resolution. These technologies have not only vastly expanded our understanding of the AML immune landscape but have also directly generated new insights into the functional regulation of NK cells. Single-cell RNA sequencing (scRNA-seq) was first introduced by Tang et al. in 2009 (22). Since then, it has been widely adopted across biological disciplines. Continuous technological innovation has since given rise to a suite of single-cell methodologies, including Smart-Seq (23), CEL-Seq (24), long-read single-cell targeted sequencing (SMRT sequencing) (25),Smart-Seq2 (26), MARS-Seq (27), Cyto-Seq (28), Drop-Seq (29), inDrop-Seq (30), CITE-seq (31), Microwell-seq (32),STRT-Seq (33), VASA-seq (34), and single-cell spatial transcriptomics (35–37). These advances provide an unprecedented perspective for dissecting the characteristics and functions of NK cells in AML.

scRNA-seq enables the unbiased revelation of transcriptional diversity in NK cells and serves as a powerful tool for refining subsets and functional states within classically defined populations. In AML research, while the foundational distinction between flow-cytometry-defined CD56^dim^ and CD56^bright^ subsets remains crucial (38), large-scale scRNA-seq studies have uncovered significant transcriptional heterogeneity and finer functional subdivisions within these populations. For instance, a study on BM NK cells from post-transplant relapsed AML patients identified an “inflammatory” transcriptional state primarily within the CD56^bright^ compartment, characterized by high expression of interferon-stimulated genes (e.g., *IFIT2*, *ISG15*). This state was enriched in relapse types accompanied by upregulated T-cell inhibitory ligands, suggesting a trajectory toward chronic activation or even functional exhaustion (39). Another study, encompassing co-cultures of 26 hematologic cancer cell lines with NK cells, systematically mapped the dynamic state transitions of NK cells upon tumor interaction. It further subdivided activated NK cells into distinct functional transcriptional modules—such as those high in genes encoding co-stimulatory/inhibitory receptors, type I interferon signaling components, or cytokines—and found that cancer cell lines, including AML, could potently induce NK cells into an activated state (40). In summary, compared to traditional flow cytometry, scRNA-seq not only enables the resolution of transcriptional heterogeneity within cell populations at an unprecedented level, allowing for the more precise delineation of novel functional subtypes, but also permits efficient preliminary validation of these potential biomarkers directly within the same experimental framework. This reduces the initial dependency on separate, targeted protein validation via flow cytometry. Consequently, these findings not only complement and extend traditional surface marker-based classifications but also fundamentally deepen our understanding of the functional transcriptional programs and continuous dynamic states of NK cells within the AML microenvironment.

To achieve a comprehensive understanding of NK cell dysfunction in AML—extending beyond conventional transcriptomic and surface-marker profiling—the investigation of the epigenetic regulatory layer is essential. Single-cell Assay for Transposase-Accessible Chromatin sequencing (scATAC-seq) offers a powerful tool for this purpose, providing unprecedented resolution into the epigenetic landscape that governs NK cell identity and function within the leukemic niche. Profiling chromatin accessibility at single-cell resolution has revealed that NK-like cells in AML exhibit a global reduction in accessibility at the promoters of activation-associated genes, directly linking this epigenetic state to their functional impairment. Moreover, integrative analyses have identified aberrant cis-regulatory elements (CREs) and disrupted transcription factor networks, involving key regulators such as *TBX21* and *RUNX3*. These networks modulate critical loci, including *CD69* and immune checkpoint genes, collectively driving NK cells toward an exhausted phenotype (41).

A systematic understanding of the phenotypic and functional complexity of NK cells in AML requires a layered, multi-omics approach. At the proteomic level, mass cytometry (CyTOF) enables deep, high-dimensional immunophenotyping of the leukemic microenvironment. In *de novo* AML, such analyses have revealed a significantly remodeled NK cell compartment. For example, a 36-parameter CyTOF study detailed the landscape of NK cell surface receptors and functional proteins, linking specific activation/inhibition states to the broader immune dysregulation (42).This protein-centric view is powerfully extended by multimodal technologies like CITE-seq, which simultaneously capture transcriptomes and surface proteomes at single-cell resolution. Such integrative profiling is key to dissecting NK cell heterogeneity, moving beyond the traditional CD56/CD16 dichotomy to define subsets with distinct cytotoxic, adaptive, or regulatory potentials. This refined taxonomy provides a precise framework for identifying AML-specific dysfunctional NK states within the tumor microenvironment (43).Crucially, the full pathophysiological meaning of these molecular phenotypes emerges only within the architecture of the complete AML ecosystem. Large-scale integrative single-cell atlases, such as one profiling over 190,000 AML cells to map cellular hierarchies and identify novel progenitor clusters, provide this essential context (44). This foundational map directs future inquiry: it allows for testing specific hypotheses on how dysfunctional NK cells interact with—and potentially fail to target—different leukemic lineages, particularly newly defined progenitor populations, thereby connecting discrete molecular signatures to systemic mechanisms of immune evasion in the BM niche.

The latest single-cell spatial omics technologies integrate spatial information with conventional single-cell sequencing data, effectively addressing the critical loss of spatial context inherent in the cell dissociation process. By providing gene expression data within intact tissue architecture, single-cell spatial omics enables precise mapping of cellular interactions and dynamic changes within the tumor microenvironment. For instance, Gege Gui et al. combined scRNA-seq with spatial transcriptomics to analyze the spatial distribution and dynamic interplay between immune cells and leukemic cells in the BM of R-AML patients after treatment with pembrolizumab (an immune checkpoint inhibitor) and the hypomethylating agent decitabine—key insights for understanding treatment response (45).Katie Maurer et al. applied multi-omics single-cell approaches (scRNA-seq, scTCR-seq, scCITE-seq) and CODEX (Phenocycler-Fusion, PCF) spatial proteomics to systematically resolve dynamic immune networks in the AML BM microenvironment. Their work highlighted the central role of ZNF683-expressing CD8+ T cells in driving responses to donor lymphocyte infusion (DLI), providing critical insights for refining cell-based therapies (46).Shovik Bandyopadhyay et al. used BM PCF panels on diagnostic (Dx) and post-treatment (PostTx) AML samples, alongside negative staging marrow (NSM) controls, and applied reciprocal principal component analysis (RPCA) to integrate multi-omics data from scRNA-seq and PCF. This integrated approach revealed shared biological variation across data modalities. Notably, myeloid cells (excluding mature subsets) were significantly increased in Dx AML compared to NSM (47.6% vs. 23.1%; p < 2.2e-16). Structural alterations were also evident—for example, near-complete loss of adipocytes in leukemic marrow—while Adipo- and THY1+ mesenchymal stromal cells were 2–3 times more frequent in AML than in NSM (47).

**Figure 1:**
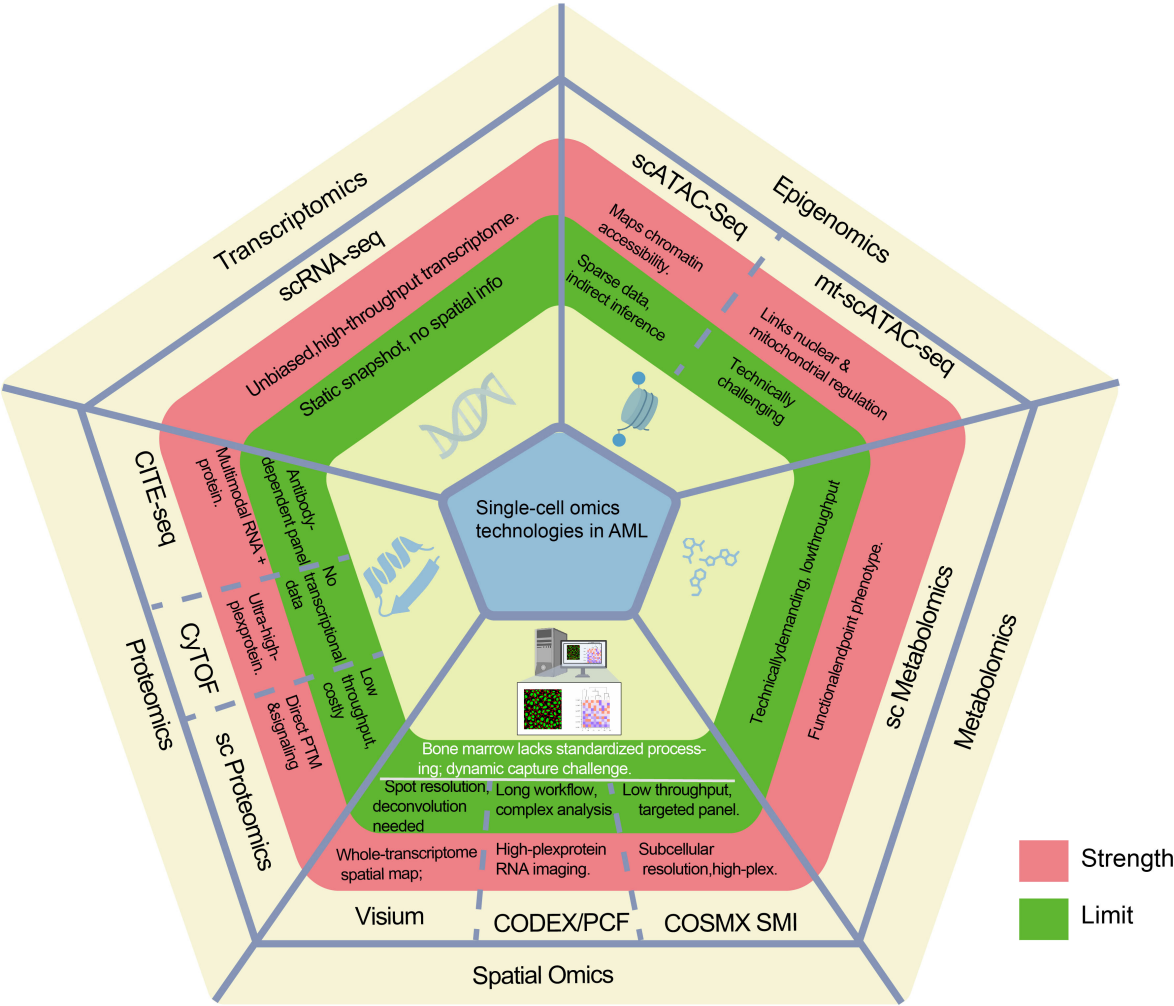
Single-cell omics technologies in AML. This figure illustrates the strengths (red) and limitations (green) of single - cell omics technologies applied in AML research, categorized into domains such as transcriptomics, epigenomics, metabolomics, proteomics, and spatial omics. Each technology within these domains is annotated with its specific advantages and constraints, offering a comprehensive overview of their roles and challenges in AML analysis.

To comprehensively decipher the functional states and regulatory mechanisms of NK cells in AML, it is imperative to integrate their dynamic molecular profiles at single-cell resolution. To this end, we first provide an overarching technical perspective by summarizing the advantages and limitations of various single-cell omics technologies currently applied in AML research (**Figure 1**) and systematically outline the specific technological platforms employed in key studies over the past five years (**Table 1**), thereby establishing a comprehensive analytical framework for a deeper understanding.

**Table 1:** Single-cell omics technologies in AML.

Single-cell omics technologies	Strengths	Limitations/constraints	Article
Single Cell Omics			
scRNA-seq	1. Resolves transcriptional heterogeneity at single-cell resolution 2. Supports high-throughput analysis (tens of thousands of cells).3. Robust, well-established cornerstone for multi-omics integration.	1.mRNA levels do not fully correlate with protein function.2. Loss of native spatial context due to tissue dissociation.3. Incomplete gene coverage due to limited capture efficiency.	(65) (66) (67) (68), (69), (70), (71), (72), (73), (74), (75), (76), (77), (78), (79), (80), (81), (82), (83), (84), (85), (47), (86), (87), (88), (89), (90), (91), (92), (93), (94), (95), (96), (97), (98), (99), (100), (101), (102), (103), (104), (46), (45), (105)
scATAC-seq	1. Maps regulatory landscapes (enhancers/promoters) and infers GRNs.2. Defines cell identity via chromatin accessibility patterns. 3. Infers TF activity through motif enrichment.	1. 1. High data sparsity and low signal-to-noise ratio.2. Accessibility is an indirect measure of transcriptional output.3. Requires specialized, complex bioinformatic pipelines.	(82) (83) (106) (107), (102), (103), (104)
mt-scATAC-seq	1. 1. Reveals regulatory causality by linking nuclear genomics to mitochondrial function. 2. Powerful tool for studying metabolism–epigenetics crosstalk.	1. Technically complex experimental and bioinformatic workflows.2. Niche applicability, primarily suited for mitochondrial biology.	(106) (104)
mtDNA-seq、	1. Detects heteroplasmy and mutations relevant to mitochondrial diseases.2. Connects mitochondrial function directly to cellular phenotypes.	Typically utilized as an add-on, not a standalone technology.	(104)
scTCR-seq	• 1. Uniquely enables precise reconstruction and tracking of T-cell clonality.2. Frequently integrated with scRNA-seq to correlate clonality with functional states.	• 1. Targeted scope; limited to TCR/BCR profiling.2. Requires integration with other omics for a complete functional view.	(108) (70) (93) (94), (97), (103), (46)
scCITE-seq,	1. Precise cell typing: Protein markers significantly enhance the accuracy of immune cell classification. 2. Functional Validation: Surface proteins directly indicate real-time function (e.g., checkpoint activity). 3. Simultaneous, multi-modal measurement of RNA and protein in the same cell. 4. Low input requirement; compatible with limited or cryopreserved clinical samples.	1. 1. Targeted design: Constrained to pre-designed antibody panels (<100 targets). 2. Higher cost relative to scRNA-seq alone. 3.Surface protein bias; largely excludes intracellular proteins.	(109) (46)
Single-cell Proteomics/Phosphoproteomics	1. PTM Detection: Capable of analyzing phosphorylation and other PTMs, revealing signaling activity. 2. Uncovers subtle, rare cell populations masked in bulk assays. 3. Provides direct, real-time functional and signaling readout.	1.Technical Challenges: Proteins cannot be amplified, demanding extreme sensitivity. 2. Lower throughput and higher expense than scRNA-seq or CyTOF. 3. Limited proteome depth (typically 1,000–3,000 proteins/cell); highly sparse data	(105)
cytometry by time-of-flight(CyTOF)	1. Ultra-High Multiplexing: Enables 40–50-plex measurement without fluorescence spillover. 2. Ultra-high throughput, ideal for analyzing millions of cells in large clinical cohorts. 3. Highly compatible with low-input clinical samples (10^4^–10^5^ cells)	1. 1. Antibody-Dependent: Limited to known epitopes and highly sensitive to antibody quality. 2.No Transcriptional Data: Captures protein levels but not dynamic gene expression.	(86)
Single-cell Metabolomics	1. 1. Functional Relevance: Metabolites represent functional endpoints, closer to phenotype than RNA. 2. Amplification-free detection, avoiding PCR biases. 3. High dynamic range; sensitive to rapid cellular responses. 3. Enables unbiased discovery and longitudinal monitoring (live-cell compatibility).	1. Technically Demanding: Metabolites are diverse, labile, and challenging to stabilize. 2. Destructive and extremely low throughput (single or few cells per run). 3. Annotation difficulties; many metabolic peaks remain unidentifiable. 4.Multi-omic data integration is computationally challenging.	(105)
Spatial Multi-omics			
CODEX	• 1. Multiplexed Spatial Analysis at single-cell resolution. 2. Compatible with FFPE (formalin-fixed, paraffin-embedded) bone marrow samples. 3. Latest systems (PCF) integrate protein and spatial transcriptomics.	1. Operational Complexity: Relies on sophisticated microfluidic reagent delivery. 2. Highly Time-Intensive ($30–50$ hours/sample) due to iterative imaging cycles. 3. High running costs and demand for advanced computational expertise	(94) (46) (47)
COMET multiplex IF	1. Delivers high-resolution protein imaging, building upon established histopathology. 2. Enables seamless integration with conventional pathology workflows.	1. Limited multiplexing capacity (constrained by fluorescence channels). 2.Requires algorithmic correction for spectral overlap. 3. Involves complex image processing and data analysis.	(110)
CosMx Spatial Molecular Imager(CosMx SMI)	1. Achieves subcellular resolution (~50–100 nm) for precise RNA localization. 2. Highly multiplexed detection of >1,000 RNAs and >60 proteins *in situ*. 3. Compatible with both FFPE and fresh-frozen clinical tissues. 4. Sequencing-free; results obtained directly via microscopy.	1. Low Throughput: Processes few tissue sections per run (days to weeks). 2. High instrument costs, typically restricted to core facilities. 3. Requires predefined gene panels, not suitable for unbiased whole-transcriptome screening.	(45)
10X Genomics Visium	1. Preserves native tissue architecture for spatially resolved transcriptomics. 2. Captures whole-transcriptome profiles, supporting novel gene discovery. 3.Visium v2 with CytAssist enables analysis of difficult decalcified bone marrow.	1.Resolution Limit: 1.55 μm spot size may encompass multiple cells, requiring deconvolution. 2. RNA degradation risk from decalcification (e.g., 10% formic acid). 3.Demands integration with scRNA-seq reference data for deconvolution.	(95) (96) (110)
GeoMx-based Digital Spatial Profiling (DSP)	1. Precise Profiling of user-defined morphological Regions of Interest (ROIs). 2. High multiplexing capacity (over 100 proteins or RNA targets). 3. Exceptional compatibility with archived FFPE tissue specimens. 4. Seamlessly integrates high-resolution imaging with multi-omic data.	1. Resolution Preclusion: Cannot achieve true single-cell resolution. 2. Technically demanding and complex workflow. 3. Destructive analysis of selected ROIs. 4. Substantial investment in instrumentation and reagents.	(96) (95)
AFADESI-MSI	1. Enables *in situ* metabolite detection at 10 μm resolution, preserving spatial context. 2. Detects diverse metabolites with broad polarity ranges. 3. Minimal sample preparation; compatible with frozen clinical sections.	1. Metabolite identification relies heavily on standardized databases. 2. Lower sensitivity for low-abundance metabolites compared to LC-MS. 3. Resolution remains limited for true single-cell level analysis. 4. Requires integration of complex spectral, spatial, and pathological data.	(111)

### 2.2 Persisting challenges and methodological frontiers

Although the integration of single-cell and spatial information offers unprecedented insights into cellular function in physiological and pathological contexts, deepening our understanding of biological systems, its specific application to deciphering the role of NK cells in AML remains relatively limited. Current data are often insufficient to fully explain the dynamic functional states, spatial localization, and cell-cell interaction networks of NK cells within the complex bone marrow microenvironment of AML. Furthermore, several technical challenges in multi-omics integration persist (48). Single-cell omics data often suffer from limited coverage, hindering comprehensive capture of molecular information and full interpretation of NK cell function and regulation. High costs restrict large-scale studies, limiting the generalizability of findings (49). These limitations are being mitigated by continuous innovation. The development of higher-throughput platforms, novel molecular capture methods, and integrated multimodal strategies are collectively pushing the boundaries. For instance, platforms employing combinatorial indexing or high-density *in situ* arrays (e.g., EasySci (50), Stereo-cell (51)) drastically reduce cost per cell and enable profiling of millions of cells, overcoming scale and coverage constraints. Concurrently, techniques that co-profile multiple molecular layers within the same cell—such as jointly measuring chromatin accessibility and gene expression (SUM-seq) or transcriptomes alongside surface protein epitopes within a spatial context (Stereo-cell-CITE) —directly address the challenge of multi-omics integration by generating inherently linked datasets (31, 51, 52). These technological leaps enhance sensitivity for rare cell states and provide a more unified molecular view.

A pivotal challenge, however, lies in integrating multi-omics data (transcriptomic, epigenomic, proteomic) to decipher the regulatory mechanisms underlying NK cell dysfunction in AML. Here, emerging tools are critical: multi-modal assays like CITE-seq and ATAC-seq, analyzed through advanced computational frameworks (e.g., Seurat, MOFA+) (53, 54), enable the direct correlation of transcriptional programs with surface protein expression or chromatin accessibility. This integration is key to mapping the coordinated molecular networks—such as those involving inhibitory receptor upregulation and metabolic dysregulation—that drive NK cell exhaustion in the leukemic niche, thereby generating actionable hypotheses for therapeutic intervention. Furthermore, sophisticated computational pipelines for batch correction and harmonization remain essential for robust data synthesis. For instance, network alignment-based integration algorithms like SCITUNA employ iterative correction strategies to output harmonized results directly in the original gene expression space, proving adept at handling the complex integration of single-cell transcriptomic and ATAC-seq datasets with varying cellular compositions (55); tools such as Crescendo perform batch correction and imputation directly on the gene expression count level, generating a corrected count matrix suitable for downstream differential analysis and specifically designed to preserve crucial spatial gene expression patterns in spatially resolved data (56).

In parallel, the application of spatial omics to hematopoietic malignancies like AML faces distinct hurdles. These cancers are often considered “liquid” with ill-defined tissue architecture, complicating sample processing and analysis. Moreover, spatial transcriptomics typically samples only limited regions of tissue, introducing sampling bias and potentially incomplete assessment of tumor microenvironment heterogeneity (47).However, recent methodological advances are beginning to address these specific limitations. Technologically, high-definition, whole-slide spatial platforms (e.g., Visium HD) offer finer resolution and larger capture areas (57). On the sample preparation front, novel wet-lab protocols for the paraffin embedding and decalcification of BM biopsies have significantly improved tissue compatibility and data quality (58). Furthermore, computational strategies such as spatial imputation and probabilistic deconvolution (59, 60)—when guided by paired single-cell RNA-seq references—can help infer cellular composition in unsampled spots and map the spatial niches of distinct cell states, thereby partially overcoming the constraint of limited sampling.

In recent years, the integration of AI with single-cell omics has injected new momentum into the study of NK cells in AML. Beyond conventional tasks like cell type annotation and outcome prediction, AI tools are proving invaluable for deciphering NK cell-specific biology. For instance, novel algorithms such as Mixed Modeling Multi-Instance Learning (MMIL), which utilizes patient-level labels to train cell-level classifiers, have shown promise in accurately identifying disease-associated cell states—a method directly applicable to pinpointing dysfunctional NK subsets, even in the absence of single-cell annotations (61). While not yet applied to AML, advanced AI-driven integration frameworks like CellMemory, which employs a bottlenecked Transformer architecture and a low-dimensional memory space to efficiently harmonize cross-platform and cross-species data with hierarchical interpretability (62), hold significant potential for constructing unified, large-scale atlases of NK cell states in the leukemic microenvironment. Similarly, end-to-end automated scRNA-seq processing systems (e.g., MLAflow)—though their use in AML remains to be fully explored—could enhance the robustness and reproducibility of NK cell cluster identification from complex datasets by leveraging Bayesian optimization to automate critical steps like hyperparameter tuning (63). Finally, dedicated image analysis software (e.g., CellDetective) (64), capable of segmenting, tracking, and analyzing NK cell dynamics in time-lapse microscopy, provides a powerful avenue for the functional validation of omics-derived hypotheses regarding NK cell-target cell interactions in AML. Collectively, these AI tools, both currently deployed and on the horizon, are poised to deepen our mechanistic understanding and accelerate therapeutic discovery.

However, the application of AI in this field comes with important limitations. First, its performance is heavily dependent on the quality, size, and precise annotation of training data, which remain scarce for specific NK cell states in AML. Second, the “black-box” nature of many complex models poses a challenge for biological interpretability. Finally, computational predictions require rigorous experimental validation. As noted in critical assessments, some user-friendly AI tools may also face limitations in scope (e.g., initially handling only 2D data) and encounter technical stability issues. Addressing these limitations—through the generation of higher-quality multimodal datasets, the development of more interpretable and robust AI architectures, and closer collaboration between computational and experimental biologists—will be crucial for realizing the full potential of AI in guiding NK cell-based therapy.

Based on the single-cell omics technological framework outlined above, the subsequent sections of this review will systematically dissect the biology of NK cells in AML. Our analysis focuses on three interrelated dimensions (1): the lineage characteristics and dynamic evolution of NK cell subsets; (2) the multi-layered etiology and molecular mechanisms driving NK cell dysfunction; and (3) the complex interaction network between NK cells and other components within the leukemic microenvironment. Together, insights derived from these fundamental mechanisms to systemic interactions will establish a cohesive theoretical foundation and highlight actionable targets for developing next-generation, NK cell-based precision immunotherapies.

## 3 Single-cell omic signatures of NK cells in the AML tumor microenvironment

### 3.1 Dysregulated distribution of NK cell subpopulations

The traditional flow cytometry classifies NK cells within the AML tumor microenvironment into the classic CD56^bright^/CD56^dim^ subsets (112). Recent studies employing high-dimensional flow cytometry, often combined with computational analyses such as principal component analysis (PCA), have moved beyond this dichotomy. They utilize an expanded marker set (e.g., CD16, CD56, CD57, CD94, KIR) to subdivide NK cells into distinct functional subsets based on their maturation stage. Key investigations have consistently identified three major maturation states in the AML setting: immature, mature/intermediate, and hypermature/terminally differentiated populations (38, 113).

While protein-centric flow cytometric approaches are excellent for phenotypic mapping, they are insufficient for fully elucidating the transcriptional and epigenetic mechanisms underlying the skewed maturation and dysfunctional receptor expression observed in AML NK cells. These limitations can be overcome by single-cell omics technologies (e.g., scRNA-seq), which transcend surface protein markers to enable high-resolution profiling across transcriptomic and epigenomic dimensions. A prime example is a study by Zhang, Zhiyong et al., which applied scRNA-seq to AML patient BM and precisely subdivided the conventional CD56^dim^ NK population. They identified distinct subsets, including AML_CD56^dim^_NK and a unique exhausted subpopulation (AML_CD56^dim^_NKex). Their findings revealed that the AML microenvironment induces a “partially exhausted” state where antitumor function is partially retained but constrained by upregulated inhibitory receptors. This biological phenotype makes them a promising target for immunotherapy, thereby directly refining the flow-cytometry-based atlas and offering a more precise molecular map for intervention (73).

Different research teams often employ distinct nomenclature for NK cell subtypes based on their specific data and objectives. Crinier, Adeline et al. categorized NK cells from AML BM into hNK_Bm1 (CD56^dim^ NK1-like), hNK_Bm2 (CD56^bright^NK2-like), hNK_Bm3 (NK0), and hNK_Bm4 (adaptive NK-like). The presence of hNK_Bm4 was mainly associated with CMV infection status rather than directly linked to AML pathogenesis. They proposed that hNK_Bm3 (NK0) serves as a precursor for both hNK_Bm1 and hNK_Bm2, with pseudotime analysis suggesting a developmental trajectory starting from hNK_Bm3 and branching into hNK_Bm1 and hNK_Bm2, while hNK_Bm4 appeared to derive from later stages of hNK_Bm1. Furthermore, they subdivided CD56^bright^ NK cells into CD56^bright^CD160^+^CD52^−^ and CD56^bright^CD160^− +^CD52^+^ subsets, noting that reduced CD160 expression in AML BM NK cells correlated with poorer survival, while high expressers showed better outcomes (65).It is crucial to interpret these pseudotime-inferred trajectories with caution. While offering a powerful, data-driven hypothesis for cellular dynamics, pseudotime analysis is an algorithmic reconstruction based on correlative transcriptional similarities within a static snapshot of cells. This “static snapshot” limitation means that the predicted developmental relationships and directionality, while insightful, remain a computational inference of potential fate rather than definitive proof of a temporal sequence. Future studies incorporating longitudinal sampling from the same patients or *in vivo* models across multiple time points, coupled with validation in larger, independent cohorts, will be essential to confirm the proposed branching trajectory from hNK_Bm3 to hNK_Bm1/hNK_Bm2 and the origin of hNK_Bm4, thereby solidifying our understanding of NK cell fate decisions in AML.

**Figure 2:**
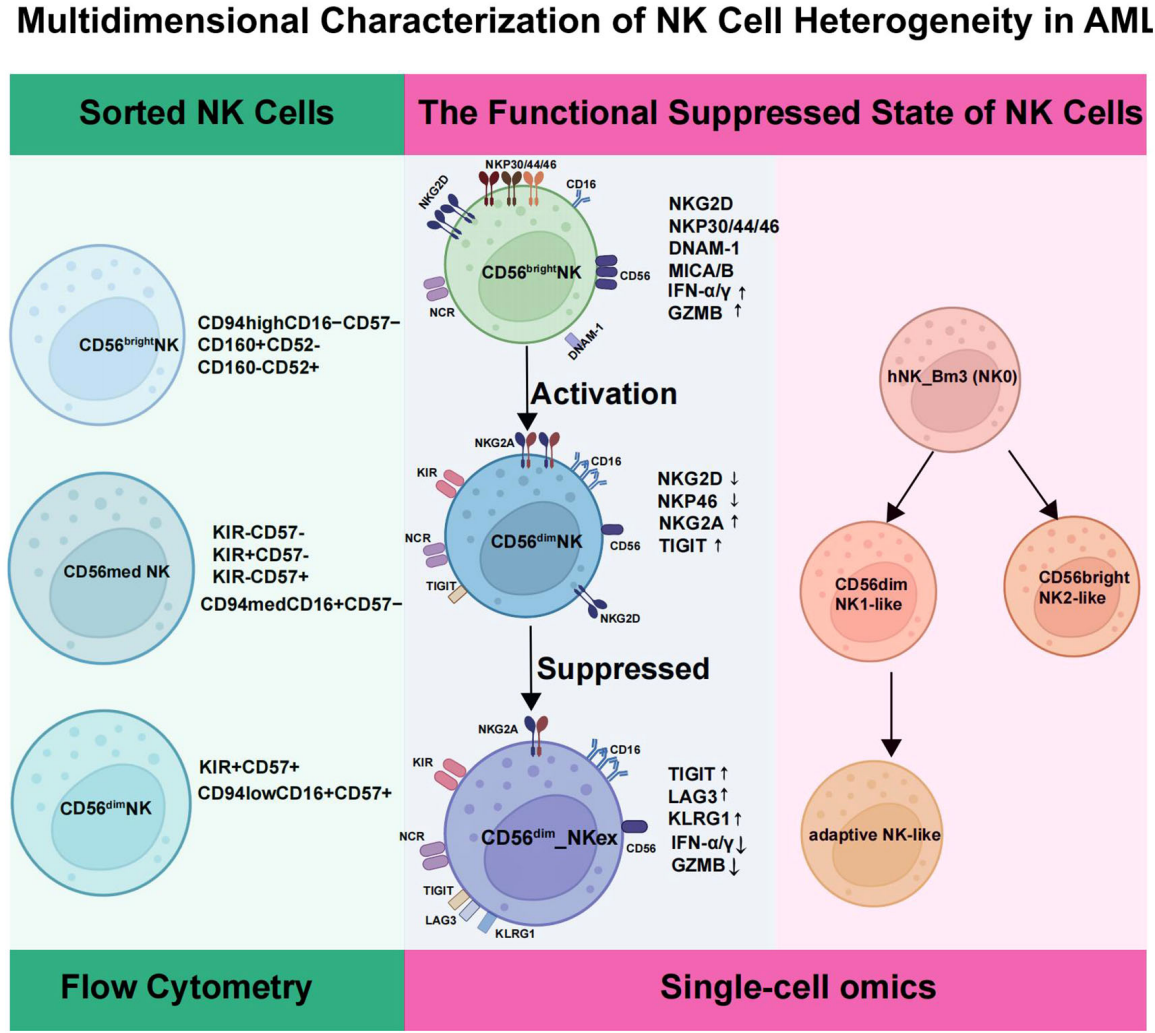
Multidimensional characterization of NK cell heterogeneity in AML. This figure integrates three complementary analytical perspectives to delineate the phenotypic, functional, and developmental landscape of NK cells within the AML microenvironment. Panel I: Phenotypic Subsets (by Flow Cytometry). NK cells are gated into three canonical subsets: CD56^bright^ (immature; CD94^high^ CD16^−^CD57^−^), CD56^med^ (transitional; CD94^med^CD16^+^CD57^−^ with variable KIR/CD57), and CD56^dim^(mature; CD94^low^CD16^+^CD57^+^KIR^+^). Panel II: Functional Pseudotime (by scRNA-seq). Pseudotime analysis orders NK cells along a continuum from an activated state (high NCRs, NKG2D, GZMB) to a mature/regulated state (reduced activation, increased NKG2A), culminating in an exhausted (NKex) state (loss of activation markers, high TIGIT/LAG3/KLRG1, low GZMB). Panel III: Developmental Trajectories (by Pseudotime). Developmental reconstruction identifies a putative progenitor (hNK_Bm3, NK0-like) giving rise to three major branches: cytotoxic (hNK_Bm1, CD56^dim^-like), regulatory (hNK_Bm2, CD56^bright^-like), and adaptive/memory-like (hNK_Bm4).

Despite variations in nomenclature across different studies, a consistent picture of NK cell dysfunction in AML clearly emerges. This dysfunction is characterized by a significant reduction in NK cell numbers and an imbalanced subset distribution, with a skew toward CD56^bright^ populations and a concomitant decrease in the highly cytotoxic CD56^dim^ subset. Systematic single-cell resolution analysis further maps these defects across multiple biological layers: from altered phenotypic profiles, through a functional continuum that progresses toward exhaustion, to the fundamentally disrupted developmental trajectories underlying these aberrant states. In summary, NK cells in AML universally exhibit profound maturational impairment and functional paralysis (**Figure 2**).

Building upon this consensus regarding phenotypic and developmental alterations, a more profound question emerges: what are the precise functional states of these finely resolved NK cell subpopulations within the immunosuppressive landscape of AML? How do their intrinsic molecular programs and interactions with the environment collectively lead to the failure of immune surveillance? Research leveraging high-resolution single-cell omics is now shifting the focus from static “phenotypic classification” to dynamic “functional deciphering,” systematically unraveling the multi-layered mechanisms underlying NK cell dysfunction.

### 3.2 NK cell dysfunction in the AML microenvironment as revealed by single-cell omics

In recent years, the emergence of scRNA-seq and related multi-omics technologies has provided unprecedented analytical power. These techniques enable systematic characterization of NK cell transcriptional programs, metabolic features, epigenetic regulation, and intercellular communication networks at single-cell resolution, thereby precisely defining states such as activation, exhaustion, dysfunction, or memory-like differentiation. It is through this high-resolution technological lens that the previously nebulous concept of “dysfunction” has been deconstructed into a series of specific, measurable abnormal states and regulatory circuits, allowing us to systematically trace and elucidate their origins.

Accordingly, this section aims to review the multi-layered, interconnected mechanisms underlying NK cell dysfunction in AML, as revealed by single-cell omics studies, focusing on the following three levels: (1) The Leukemic Cell Level: How genetic or transcriptional alterations affecting HLA expression, resolved by single-cell multi-omics technologies, render target cells “unrecognizable” to NK cells; (2) The Intrinsic State of NK Cells Level: How key signals, revealed by single-cell transcriptome analysis of the tumor microenvironment, directly induce a functionally suppressed state in NK cells; (3) The Systemic Interaction Network Level: How broader cytokine signaling and intercellular dialogues, elucidated by single-cell communication analysis, synergistically disrupt NK cell function, collectively contributing to immune escape.

#### 3.2.1 Impaired HLA expression

In normal immune surveillance, human leukocyte antigen (HLA) class I molecules on cell surfaces engage inhibitory receptors (such as killer cell immunoglobulin-like receptors, KIRs) on NK cells, delivering a “self” signal that prevents attack on healthy host cells. This delicate balance is fundamental for NK cells to distinguish “self” from “non-self.” However, in AML, leukemic cells disrupt this equilibrium through multiple mechanisms, actively cloaking themselves and leaving NK cells in a state of “non-recognition.”

The traditional view holds that tumor cells might evade cytotoxic T cell recognition by completely losing HLA expression, but this would conversely release inhibition on NK cells, triggering “missing-self” killing. Therefore, AML cells more frequently employ a more refined and cunning strategy: they do not simply lose HLA entirely but instead undergo partial allelic loss, transcriptional downregulation, or epigenetic silencing, leading to abnormally low or altered HLA expression. This “disguise” is insufficient to effectively activate T cells yet significantly weakens or interferes with their ability to deliver inhibitory signals to NK cells.

Single-cell multi-omics technologies, particularly the integrated analysis of scRNA-seq and single-cell assay for scATAC-seq, provide unprecedented resolution for dissecting this complex “cloaking” mechanism. Through scRNA-seq, researchers can directly observe significant heterogeneity in the transcript abundance of HLA genes (e.g., *HLA-A, -B, -C, -E*) at the resolution of individual leukemic cells and identify which AML cell subpopulations possess specific HLA-low expression signatures. More importantly, combined with scATAC-seq data, one can trace the potential origin of this transcriptional alteration: for instance, discovering reduced chromatin accessibility in the promoter or enhancer regions of HLA genes, or identifying dysregulation of associated specific transcription factor networks (e.g., impaired activity of interferon regulatory factors, *IRFs*). These findings transform the abstract notion of “downregulation” into concrete, localizable epigenetic abnormalities (85, 114, 115).

Furthermore, multiplexed single-cell sequencing technologies (e.g., NAMUL-seq, which simultaneously detects transcriptome and genome) help reveal somatic genetic alterations affecting HLA, such as identifying chromosome region deletions (e.g., loss of the short arm of chromosome 6) or specific mutations associated with HLA loss at the single-cell level. These genetic events, together with the aforementioned transcriptional/epigenetic regulatory abnormalities, form the multi-layered foundation for AML cells to evade NK cell recognition.

Critically, the application of scRNA-seq fundamentally overcomes the limitations of traditional bulk sequencing. Bulk sequencing cannot distinguish whether changes in HLA expression stem from intrinsic transcriptional reprogramming within leukemic cells or merely reflect fluctuations in the immune cell composition of the sample. In contrast, scRNA-seq, at single-cell resolution, can definitively identify those subpopulations of leukemic cells with specific HLA-low expression signatures, confirming this as an active, cell-intrinsic immune evasion program. In the future, integrating long-read sequencing technologies with spatial transcriptomics will not only allow more precise quantification of HLA expression at the allelic level but also enable the mapping of spatial correlations between different leukemic clones and HLA loss patterns within the three-dimensional context of the native BM, ultimately providing a complete, fine-grained map of AML immune escape at single-cell, allelic, and spatial dimensions.

In summary, AML leukemic cells do not passively remain “ignored”; they actively exploit complex alterations at the genetic, epigenetic, and transcriptional levels to finely tune their HLA expression landscape. Single-cell multi-omics technologies act like a high-precision “molecular microscope,” revealing these abnormalities one by one that were previously masked by population-averaged analyses. They clearly elucidate the first key reason for NK cells’ “inability to recognize” their targets: the inhibitory signal reception end (i.e., the HLA-KIR axis) is purposefully weakened or disrupted by the leukemic cells. This precisely targeted immune evasion strategy provides crucial scientific rationale for developing therapeutic interventions aimed at restoring or mimicking normal HLA expression (e.g., epigenetic drugs).

#### 3.2.2 The functional suppressed state of NK cells

The advent of scRNA-seq and related multi-omics technologies has revolutionized our understanding of NK cell biology in AML. Moving beyond bulk analyses that mask cellular heterogeneity, these approaches allow for the precise dissection of NK cell subsets, functional states, dynamic transitions, and cell-cell communication networks within the leukemic microenvironment. The following section synthesizes key findings on NK cell dysfunction, highlighting how single-cell omics have been instrumental in defining these pathological states.

Multiple studies have demonstrated that NK cells possess the potential to induce durable responses in AML. Unlike T or B cells, NK cells are antigen-unrestricted and do not trigger autoimmune reactions or graft-versus-host disease (GVHD). As a result, NK cells represent a safer and more readily available ‘ready-to-use’ allogeneic product for patients. Their functional state is highly dependent on a precise balance within the network of surface receptors: activating receptors (such as NKG2D, NKp30, and DNAM-1) initiate killing programs, while inhibitory receptors (such as NKG2A, KIR, and TIGIT) recognize self MHC-I molecules to prevent excessive responses. The balance between these opposing signals determines the cytotoxic activity of NK cells (38, 116).

However, while traditional research has established that NK cell function is impaired in AML patients, the understanding of their intrinsic state remained superficial. scRNA-seq technology has fundamentally altered this landscape by revealing profound functional heterogeneity and a continuum of states within the NK cell population. Through unsupervised clustering and trajectory inference analyses, studies have definitively identified NK cell subpopulations that are significantly expanded in the AML microenvironment and bear specific molecular signatures. These cells are not simply “inactive” but have entered a finely programmed dysfunctional state. The core features of this state, precisely captured by single-cell data, are primarily manifested at three levels:

First, there is the dominant expression of inhibitory checkpoints and the establishment of key signaling axes. scRNA-seq data show that within the AML BM, particularly in the CD56bright NK cell subset—which typically possesses immunoregulatory functions and is found to be relatively enriched—the transcription level of the inhibitory receptor NKG2A (*KLRC1/CD159a*) is significantly upregulated. Its ligand, HLA-E, is highly expressed on the surface of blasts in relapsed/refractory AML, forming a high-affinity HLA-E–NKG2A complex. Single-cell communication analysis (e.g., CellChat) confirms the significant enrichment of this inhibitory axis within the microenvironment. Upon ligation, the intracellular ITIM motifs of NKG2A rapidly recruit the protein tyrosine phosphatases SHP-1 and SHP-2, which dephosphorylate PI3K, thereby inhibiting the *PI3K–AKT–mTORC1* signaling pathway. This signal attenuation directly blocks cytotoxic granule polarization and exocytosis, leading to impaired NK cell degranulation and function (36, 82, 117). This is not an isolated phenomenon. Single-cell analysis further reveals a co-expression pattern of multiple co-inhibitory receptors—such as *TIGIT*, *LAG-3*, *TIM-3*, and *KLRG1*—on the same NK cell subpopulation. This pattern aligns with descriptions of an exhausted NK cell phenotype in peripheral blood (PB) and BM (accompanied by impaired production of IFN-γ and granzyme B) (118), collectively defining an exhaustion-like phenotype. This molecular signature of “multiple brakes” is a direct consequence of chronic antigen stimulation and inhibitory microenvironmental signals, the sources of which can be traced through computational communication analysis.

Second, activating receptors are downregulated, and effector function transcriptional programs are comprehensively suppressed. Comparative single-cell transcriptome analysis shows that, compared to healthy controls, key activating receptors are significantly downregulated in NK cells from AML patients. These include NKG2D (its expression is markedly suppressed in the AML microenvironment, and its ligands MICA/B are often downregulated by leukemia cells), the NCR family (*NKp30*, *NKp44*, *NKp46*) (where NKp46 expression is reduced in patients, and NKp30 can be downregulated by stromal cells via TGF-β1 and IL-10), and *CD226 (DNAM-1)* (its downregulation is induced by aberrant overexpression of CD155 on tumor cells, leading to its internalization and ubiquitin-dependent degradation). Concurrently, the expression of genes encoding critical cytotoxic mediators (e.g., granzyme B/*GZMB*, perforin/*PRF1*) and effector cytokines (e.g., interferon-γ/*IFNG*, tumor necrosis factor-α/*TNF*) is also significantly reduced (68, 78, 119). This widespread “deactivation” state results from multiple microenvironmental mechanisms, such as suppression of NCR expression by factors like TGF-β and IL-10, internalization and degradation of CD226 induced by CD155 overexpression, and direct induction of exhaustion via the Galectin-9/TIM-3 axis (108). Computational methods like Gene Set Variation Analysis (GSVA) quantify the decreased activity scores of pathways related to cytotoxicity and IFN-γ response. Furthermore, single-cell regulatory network inference (e.g., pySCENIC) analysis reveals that the IFN-α/γ response pathway and its associated transcription factors are significantly downregulated in NK cells from AML patients compared to healthy donors (70, 73, 87, 90). This indicates that inhibitory signal input has successfully reprogrammed the core transcriptional program of NK cells, depriving them of the essential tools for executing killing functions.

Finally, there is adaptive reprogramming of metabolism and survival pathways. In-depth single-cell analysis also finds altered metabolic states in functionally suppressed NK cells. Signaling from inhibitory receptors (e.g., NKG2A) directly attenuates the PI3K-AKT-mTORC1 pathway, which not only affects effector function but also profoundly alters cellular metabolism and survival. Additionally, the AML microenvironment exerts metabolic pressure through soluble factors (e.g., PGE2), inducing hyperactivation of the *cAMP-CREM* signaling axis within NK cells. This subsequently leads to phosphorylation-mediated blockade of the *LCK-ERK* pathway, inhibiting cytotoxicity (120). These changes likely impair metabolism-related pathways associated with sustained vitality and function (e.g., oxidative phosphorylation) while activating certain stress- or exhaustion-related metabolic patterns. This metabolic reprogramming is consistent with the inhibitory signals from surface receptors and downstream transcriptional alterations, collectively maintaining the NK cells in a low-function state.

In summary, the AML tumor microenvironment does not act through a single signal but via an interwoven signaling network—including the remodeling of ligands on leukemia cell surfaces (e.g., upregulation of HLA-E, Galectin-9, CD155; downregulation of MICA/B), soluble factors (e.g., TGF-β, IL-10, PGE2), cell contact-dependent signals (e.g., HLA-E/NKG2A, Gal-9/TIM-3), and metabolic pressure (e.g., the cAMP pathway)—that continuously acts on NK cells. The key contribution of technologies like scRNA-seq, GSVA, and communication inference lies in their ability to directly link these complex, multi-layered microenvironmental inputs to precise molecular state changes within NK cells (receptor expression imbalance, effector program silencing, signaling pathway inhibition, metabolic reprogramming). This provides single-cell resolution evidence that the tumor microenvironment, by actively sending a series of inhibitory signals, directly induces a systematic reprogramming of the intrinsic transcriptional state and functional potential of NK cells, locking them into a multi-facet, functionally suppressed, exhaustion-like, or dysfunctional stable state. This represents the core cell-intrinsic mechanism enabling immune escape in AML. This understanding provides direct molecular targets and a rationale for developing therapies to reverse the NK cell functional state, such as blocking checkpoints like NKG2A and TIGIT, using IL-15 superagonists, inhibiting MICA/B shedding to enhance NKG2D recognition, and applying CAR-NK cells that can bypass intrinsic receptor defects (98).

Recent studies utilizing a combination of flow cytometry, single-cell multi-omics sequencing, and advanced spatial analysis techniques, coupled with rigorous *in vivo* and *in vitro* validation models, have deepened our understanding of NK cell functional abnormalities and their therapeutic potential in AML from multiple dimensions. Research finds that NK cells in AML patients not only exhibit functional exhaustion and maturation blockade, but their state also serves as an important independent prognostic indicator. For instance, unsupervised clustering via *in vitro* flow cytometry, combined with *in vitro* functional validation experiments, can categorize patient NK cells into groups with distinct maturation characteristics based on the expression of CD56, KIR, and CD57. Clinical cohort analysis (*in vivo* evidence) further shows that patients exhibiting an early maturation blockade phenotype have significantly worse prognoses, and the frequency of anti-leukemic potential memory-like NK cells (CD56dim/CD57+/NKG2C+) is also lower within this group. This aligns with and corroborates the “exhaustion-like” state and receptor imbalance revealed by prior single-cell omics, collectively indicating that intrinsic NK cell functional defects are a key, quantifiable clinical target in AML immune microenvironment dysregulation.

To reverse this functional suppression, novel therapeutic strategies are being developed to precisely target the aforementioned abnormal states. On one hand, progress has been made in immunomodulatory strategies aimed at “reprogramming” or “reviving” existing endogenous NK cells. For example, clinical studies (*in vivo* validation) using the aAVC-WT1 vaccine platform demonstrate that this therapy can effectively activate dysfunctional NK cells and iNKT cells in RR-AML patients, restoring their ability to produce IFN-γ, thereby reversing the suppressive microenvironment and initiating subsequent immune responses. *In vitro* co-culture experiments further confirm the enhanced killing capacity of vaccine-activated NK cells against leukemia cells, providing mechanistic support. On the other hand, “replacement” strategies that directly provide new effector cells show breakthrough potential. Through clinical infusion studies (*in vivo* validation) combined with high-dimensional analysis, employing mass cytometry (CyTOF) and single-cell transcriptomic/proteomic sequencing, it has been confirmed that in the setting of immunocompatible haploidentical transplantation, infusion of donor-derived cytokine-induced memory-like (ML) NK cells can achieve over a thousand-fold expansion *in vivo* and functionally persist for more than 60 days in patients. Further *in vitro* functional analysis shows that these ML NK cells not only successfully engraft but also exhibit unique activated epigenetic and transcriptional signatures; their cytotoxic function in *in vitro* killing assays is significantly superior to that of the patient’s residual, suppressed NK cells.

These advances collectively outline a clear paradigm shift: from traditional non-specific immune stimulation towards interventions based on deep molecular profiling that precisely target specific NK cell dysfunctional states. Whether through vaccines to relieve suppression and reprogram endogenous cells or through direct adoptive transfer of engineered, robust effector cells, the core lies in directly repairing or replacing the failed NK cell functional units. These strategies have all undergone complete research pipelines from *in vitro* mechanistic validation to *in vivo* efficacy evaluation, marking the targeting of NK cell functional state as a key frontier direction for overcoming AML immunosuppression and improving treatment efficacy, laying the foundation for future combined strategies incorporating microenvironment modulation.

#### 3.2.3 Dynamic ecosystem of the AML immune microenvironment as resolved by single-cell and spatial Omics

Traditional research, constrained by technological resolution, has struggled to systematically uncover the global landscape of multicellular interactions within the BM microenvironment of AML. By leveraging scRNA-seq, single-cell assay for scATAC-seq, and the derived cell communication inference analyses (e.g., CellChat, NicheNet), combined with emerging Spatial Transcriptomics and Multiplexed Immunofluorescence (IF) techniques, we have moved beyond previous static, population-level observations. For the first time, we can systematically delineate, at single-cell precision and spatial *in situ* resolution, a highly dynamic and plastic immunosuppressive ecosystem that is actively orchestrated by leukemic cells. This ecosystem collaboratively induces the functional paralysis of NK cells across multiple dimensions.

Integrated scRNA-seq and scATAC-seq analyses indicate that AML cells are not passive adapters to their environment but can actively remodel the surrounding BM mesenchymal stromal cells (MSCs). Research reveals that leukemic cell subsets, particularly those harboring specific genotypes such as t (8, 21), can induce an imbalance in MSC differentiation towards pro-inflammatory and immunosuppressive phenotypes through *GATA* family transcription factor-mediated epigenetic reprogramming. Ligand-receptor interaction analysis (e.g., CellChat) further confirms that these “educated” MSCs establish intimate bidirectional communication with leukemic cells and transform into hubs that constitutively secrete multiple immunosuppressive factors (103). These factors include PGE2 and TGF-β, which directly impair NK cell cytotoxic signaling, and *IDO*, which indirectly suppresses immunity by depleting tryptophan and promoting regulatory T cell expansion. Moreover, MSC-derived cytokines such as *IL-6* and *GM-CSF* have been shown to promote the differentiation of myeloid-derived suppressor cells (MDSCs), thereby demonstrating the cascading amplification effect of the inhibitory network at single-cell resolution.

Deep clustering analysis of dendritic cells (DCs) uncovers a functional paradox in AML-associated DCs. Their highly expressed and secreted *S100A8/A9* proteins are observed at the single-cell level to coincide with *IL-6* production in NK cells. However, this DC subset is simultaneously significantly correlated with NK cell exhaustion gene signatures. More critically, cell communication analysis clearly shows a strong predicted interaction signal between the highly expressed *PD-L1* on DCs and *PD-1* on NK cells, providing computational evidence for the PD-1/PD-L1 axis directly mediating NK cell exhaustion.

Comparative analysis of scRNA-seq data from healthy individuals and AML patients reveals a rupture in the cooperative mechanism between NK cells and cytotoxic T lymphocytes (CTLs). Specifically, the expression of the co-stimulatory molecule CD244 on NK cells is generally downregulated, whereas CD8^+^ T cells are enriched for exhaustion-associated genes like *TIGIT* and *LAG-3*. This “dual incapacitation” state, observed at the single-cell level, explains their failure to collaborate effectively via the *CD48-CD244* pathway and the *IFN-γ/IFNGR1* axis (87, 114).

Re-subtyping studies on innate lymphoid cells (ILCs) demonstrate that the AML microenvironment skews ILC differentiation trajectories, leading to an abnormal increase in the proportion of the ILC3 subset, which possesses potential pro-tumor or immunoregulatory functions. Trajectory inference analysis suggests these ILC3s may compete with NK cells for limited cytokines or niche resources, thereby weakening innate immune surveillance from within.

Integrated analysis of Spatial Transcriptomics and scRNA-seq provides a direct visualization of the abnormal physical distribution patterns of immune cells within the AML BM. Studies find that during relapse, leukemic cells downregulate *CXCR4* expression to detach from CXCL12-high, immune-enriched niches. Concurrently, the *CXCL8* signal responsible for recruiting NK cells exhibits a spatially weakened or disordered pattern. This spatial “isolation” mechanism is difficult to capture with conventional sequencing approaches (121).

By correlating scRNA-seq data with metabolomics or metabolic flux analysis, researchers have discovered that MSCs can secrete metabolites like taurine, which, via the TAUT transporter, activates the mTORC1 signaling and glycolytic pathway in leukemic stem cells, directly providing metabolic support. On the other hand, single-cell data confirm that post-chemotherapy AML cells highly express CD39. Its mediated conversion of ATP to adenosine creates a local high-adenosine immunosuppressive microenvironment that directly inhibits NK cell function. This coupling of metabolic reprogramming with immune suppression is a mechanism that can only be clearly elucidated following the resolution of microenvironment heterogeneity by single-cell technologies (46, 47, 94).

In summary, the integration of single-cell transcriptomics, epigenomics, cell communication analysis, and spatial multi-omics technologies enables the construction of a three-dimensional immunosuppressive network model. This model centers on the core interactive unit of leukemic cells and MSCs and encompasses the participation of various other cells, including DCs, exhausted T cells, MDSCs, and ILC3s. It systematically elucidates the biological basis for the cooperative suppression of NK cell function through multidimensional mechanisms involving soluble factors, membrane-bound checkpoints, metabolites, and chemokine signals (95, 96).

However, most current mechanistic studies remain at the level of molecular associations, and two core questions urgently await answers: First, what are the precise spatial localization and interaction patterns of these functionally suppressed immune cells within the three-dimensional bone marrow space? Spatial multi-omics technologies (such as multiplex immunofluorescence and spatial transcriptomics) have preliminarily revealed that AML bone marrow is not an immune “desert” but may harbor abnormal tissue structures (such as tertiary lymphoid structure-like aggregates associated with poor prognosis). Future research needs to clarify the distribution and state of NK cells within these specific spatial regions. Second, how does the microenvironment dynamically drive drug resistance and relapse? Multi-omics studies indicate that relapse following chemotherapy often stems from epigenetic reprogramming and cellular state plasticity of leukemic cells (e.g., acquisition of stem cell-like properties), rather than from newly acquired driver mutations. Furthermore, cell type-specific mutations in non-coding regions may regulate this process by affecting enhancer activity.

**Figure 3:**
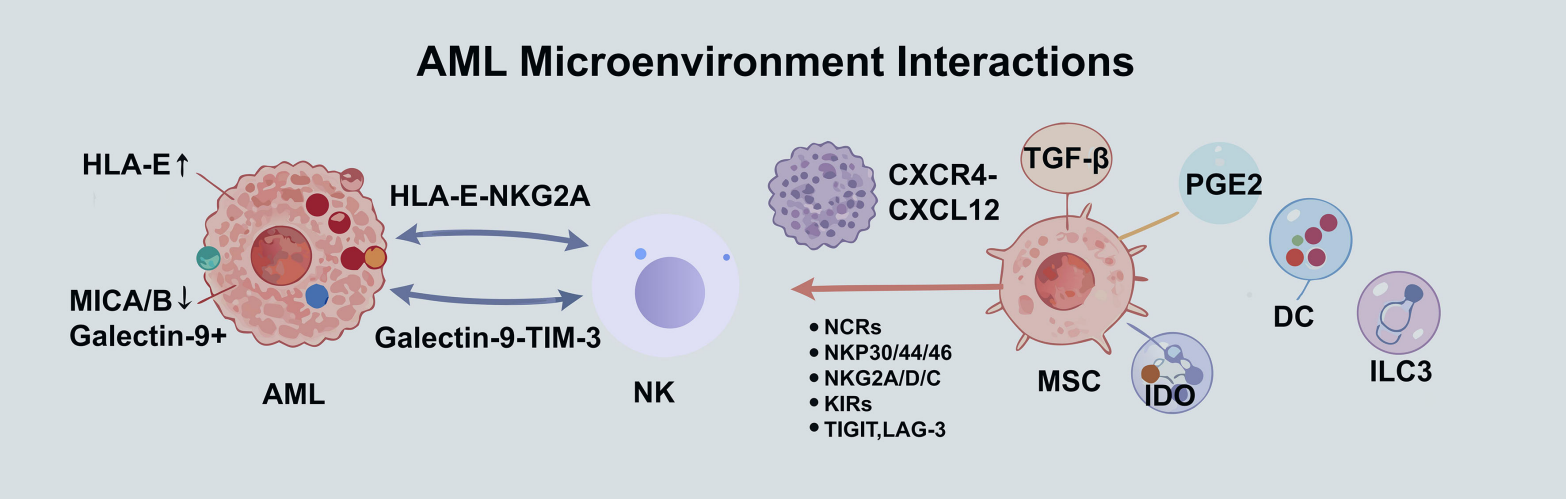
Cellular interaction mechanisms in the AML microenvironment. This figure illustrates the key molecular interactions between AML cells and immune/stromal cells within the bone marrow niche that facilitate immune evasion and disease progression. 1. AML-NK Cell Interactions: AML cells evade NK cell surveillance by upregulating inhibitory ligands (HLA-E, Galectin-9) that engage NK cell receptors (NKG2A, TIM-3), while often downregulating activating ligands like MICA/B (for NKG2D). 2. Role of Mesenchymal Stromal Cells (MSCs): MSCs recruit AML cells via CXCL12-CXCR4 and secrete immunosuppressive factors (TGF-β, PGE2). These factors suppress NK cell activating receptors (NCRs, KIRs) and upregulate inhibitory receptors (TIGIT, LAG-3). 3. Broader Immunosuppressive Network: MSCs further modulate other immune cells (e.g., DCs, ILC3s) via molecules like IDO, collectively fostering a microenvironment that supports AML progression and immune escape.

Consequently, these high-resolution technologies have catalyzed a fundamental paradigm shift in therapeutic strategy: from targeting single nodes for mere “activation” or “blockade” towards the “bidirectional remodeling” of the entire tumor ecosystem. Future treatment approaches must combine two facets: first, the sequential activation or redirection of immune effector functions (e.g., employing CAR-NK cells); and second, the simultaneous dismantling of the microenvironment’s metabolic and physical support for leukemic cells alongside its active suppression of immune cells (e.g., targeting the inhibitory secretions of MSCs, blocking the uptake of key metabolites like taurine, or intervening in the epigenetic plasticity of leukemic cells). By leveraging the systematic blueprint provided by integrated single-cell, spatial, and dynamic temporal analyses, we can aspire to achieve precise deconstruction and fundamental reconstruction of the AML immune microenvironment, thereby attaining long-term disease control.(**Figure 3**).

## 4 Cell-based therapy

As critical effectors of innate immunity, NK cells have attracted widespread attention for their anti-tumor activity in AML cell-based therapy (20). However, the immunosuppressive nature of the AML tumor microenvironment often impairs NK cell function, leading to exhaustion and reduced cytotoxicity. Single-cell omics technologies now offer precise molecular insights to optimize NK cell-based therapeutic strategies. The following sections discuss recent advances in NK cell therapy for AML guided by single-cell analyses, covering both conventional NK cell therapies and emerging CAR-NK approaches.

### 4.1 Conventional NK Cell therapies

Conventional NK cell therapies aim to enhance anti-leukemic activity by modulating NK cell function, counteracting immunosuppressive signals, or combining with other treatments. Single-cell omics has revealed functional characteristics of NK cells and their association with treatment efficacy under various intervention strategies (122).

#### 4.1.1 NK cells infusion

Single-cell data highlight how NK persistence and metabolic fitness determine infusion. Transfer of donor-derived NK cells represents a classical AML treatment strategy. Its efficacy is closely linked to the persistence and functional state of NK cells *in vivo*. Single-cell transcriptomic analyses revealed that *IL-15*/*IL-12*/*IL-18*-pretreated “memory-like NK cells” can persist for more than two months in immunocompatible AML patients (109). These cells exhibit elevated expression of proliferation-related genes (e.g., *MYC*) and metabolic activity genes (e.g., glycolytic pathway genes), which correlate directly with enhanced anti-tumor activity (123). Furthermore, peripheral blood-derived CD56dim NK cells demonstrate superior cytotoxicity compared to umbilical cord blood-derived CD56bright subsets, owing to higher expression of cytotoxic molecules (*PRF1*, *GZMB*). Single-cell sequencing further confirmed enrichment of “activation–cytotoxicity” pathway genes (e.g., *IFNG*, *TNF*) in the CD56dim subset, providing a molecular rationale for cell source selection (19, 123).These findings underscore that pre-activation status and metabolic programming are key determinants of *in vivo* NK cell persistence and cytotoxicity.

#### 4.1.2 Immune checkpoint blockade

scRNA-seq provides a molecular rationale for tailoring checkpoint inhibitors to baseline NK subset composition. As noted, NK cells in AML patients often overexpress immune checkpoint molecules (e.g., *NKG2A*, *TIGIT*), contributing to functional exhaustion. scRNA-seq revealed significant upregulation of *NKG2A* (*KLRC1*) in NK cells from relapsed AML patients, accompanied by suppression of the *PI3K–AKT* pathway. Blocking *NKG2A* or genetically ablating *KLRC1* restored AKT phosphorylation and cytotoxic function-a mechanism validated both *in vitro* and in AML mouse model (124). Additionally, patients responding to anti-TIM3 or anti-PD1 therapy exhibited higher proportions of mature NK cells (CD56dim and memory-like subtypes) at baseline. After treatment, these patients showed a greater number of differentially expressed genes (e.g., *IFNG*, *CCL4*) in NK cells compared to those receiving anti-CTLA4, suggesting that checkpoint inhibitor selection should account for baseline NK cell subset characteristics (93).scRNA-seq not only identifies actionable immune checkpoints but also informs patient stratification based on baseline NK cell characteristics. Moreover, applying single-cell profiling for longitudinal disease monitoring is crucial, as it captures time-dependent changes in receptor expression. By tracking the dynamic downregulation of exhaustion markers (e.g., *TIGIT*, *TIM3*) during treatment, clinicians can evaluate therapeutic efficacy in real-time and adjust regimens before clinical relapse occurs.

#### 4.1.3 Conventional chemotherapy

Single-cell transcriptomics reveals that chemotherapy induces dynamic, time-dependent reshaping of NK immunity, emphasizing the critical need for longitudinal single-cell monitoring throughout the treatment course. Cytarabine can trigger NK cell apoptosis and suppress cytotoxic programs, depleting effector molecules like *PRF1* and *GZMB*( *(*81). Yet, a counterintuitive expansion of NK cells is frequently observed in non-remission patients. These residual cells adopt a distinct immunosuppressive phenotype, defined by high *S100A8*/*S100A9* expression and linked to oxidative stress and autophagy. This single-cell-mapped reprogramming reveals a tangible mechanism of chemoresistance, positioning NK cell state heterogeneity as a compelling therapeutic target (125).Thus, chemotherapy exerts dual effects on NK cells—depleting effector populations while fostering immunosuppressive subsets—highlighting the need for therapies that counteract this detrimental reprogramming. This highlights the importance of analyzing time-dependent NK changes: continuous profiling can pinpoint the precise therapeutic window where NK cells transition from an effector to a suppressive state, enabling timely interventions to reverse this detrimental reprogramming.

#### 4.1.4 Targeted therapy and combination strategies

Single-cell omics not only uncovers individual therapeutic targets but also provides a mechanistic framework for rational combination strategies. For instance, scRNA-seq analysis of the AML tumor microenvironment revealed a significant loss of NKT cells in high-risk cases, prompting the identification of *PLA2G4A* as a metabolic checkpoint. Inhibiting this enzyme was shown to enhance NK cytotoxicity by upregulating the *NKG2D* receptor, a mechanism confirmed via single-cell resolution (126). Furthermore, gene regulatory network analysis demonstrated that *c-JUN* inhibition suppresses *CD33* expression, thereby increasing AML susceptibility to immune attack. This insight facilitated a synergistic combination therapy using dual *c-JUN*/*HDAC1* inhibition to activate “killing–inflammatory” pathways in NK cells (127).

However, transcriptomic changes are often driven by deeper epigenetic modifications. Deciphering the AML epigenomic landscape through scATAC-seq provides a more fundamental understanding of NK cell dysfunction by mapping chromatin accessibility. Specifically, scATAC-seq has identified key dysregulated loci—such as the promoters of activation-related genes and immune checkpoint regions—that are epigenetically silenced in the AML environment.

This molecular blueprint directly informs the use of epigenetic drugs to “prime” the immune system. For example, DNMT inhibitors (e.g., azacitidine) and HDAC inhibitors (e.g., vorinostat) can reverse these aberrant chromatin states. Mechanistically, azacitidine sensitizes leukemic cells by upregulating NK-activating ligands, while vorinostat directly targets the identified dysregulated loci to restore NK cell effector function and cytotoxicity (114, 128). By integrating scRNA-seq and scATAC-seq, clinicians can now design precision combination regimens that simultaneously overcome metabolic suppression and epigenetic silencing.

### 4.2 CAR-NK cells therapy

Conventional NK-cell-based approaches modulate function or the microenvironment to boost anti-tumor activity, their success is constrained by AML heterogeneity. While conventional NK therapies rely on exogenous activation, next-generation CAR-NK cells integrate antigen-specific recognition directly into the NK effectors. The emergence of CAR-NK therapy marks a move away from external modulation towards intrinsic precision engineering, granting NK cells specific targeting capability through chimeric antigen receptors for more accurate leukemic cell recognition and clearance. CAR-NK therapy involves genetically engineering NK cells to express CARs that recognize tumor-specific antigens. This strategy involves genetically engineering NK cells to express receptors that recognize tumor-associated antigens, effectively combining antigen-specific targeting with innate NK cell cytotoxicity. Upon antigen engagement via single-chain variable fragments, CAR-NK cells become activated and mediate precise elimination of leukemia cells (129–131).

#### 4.2.1 Advantages of CAR-NK therapy

CAR-NK cells present unique advantages over conventional NK cell therapies. Their targeting specificity is significantly enhanced: while conventional NK cells rely on natural receptors (e.g., *NKG2D*) for target recognition-often compromised by AML downregulation of ligands like MICA/B-CAR-NK cells directly bind antigens such as *CD33 (*120), increasing killing rates against AML cell lines (THP-1, U937) by 30–50% compared to unmodified NK cells (132, 133). They also exhibit greater resistance to immunosuppressive microenvironments: conventional NK cells are susceptible to inhibition by PGE2 and TGF-β in the BM, resulting in decreased cytotoxicity (*PRF1*, *GZMB*) *(*125), whereas CAR constructs incorporating 4-1BB costimulatory domains activate the PI3K–AKT pathway, enabling the cells to maintain robust cytokine secretion (IFNG, TNF) even in hostile environments (123, 129). Additionally, CAR-NK cells demonstrate superior persistence: while traditional NK cells survive less than two weeks *in vivo*, iPSC-derived engineered NK cells have demonstrated functional persistence for over four weeks in AML models while sustaining peak cytotoxic activity (134).These advantages position CAR-NK cells as a promising therapeutic modality capable of overcoming multiple limitations of conventional NK cell therapies.

#### 4.2.2 Overcoming clinical bottlenecks via single-cell insights

**Figure 4:**
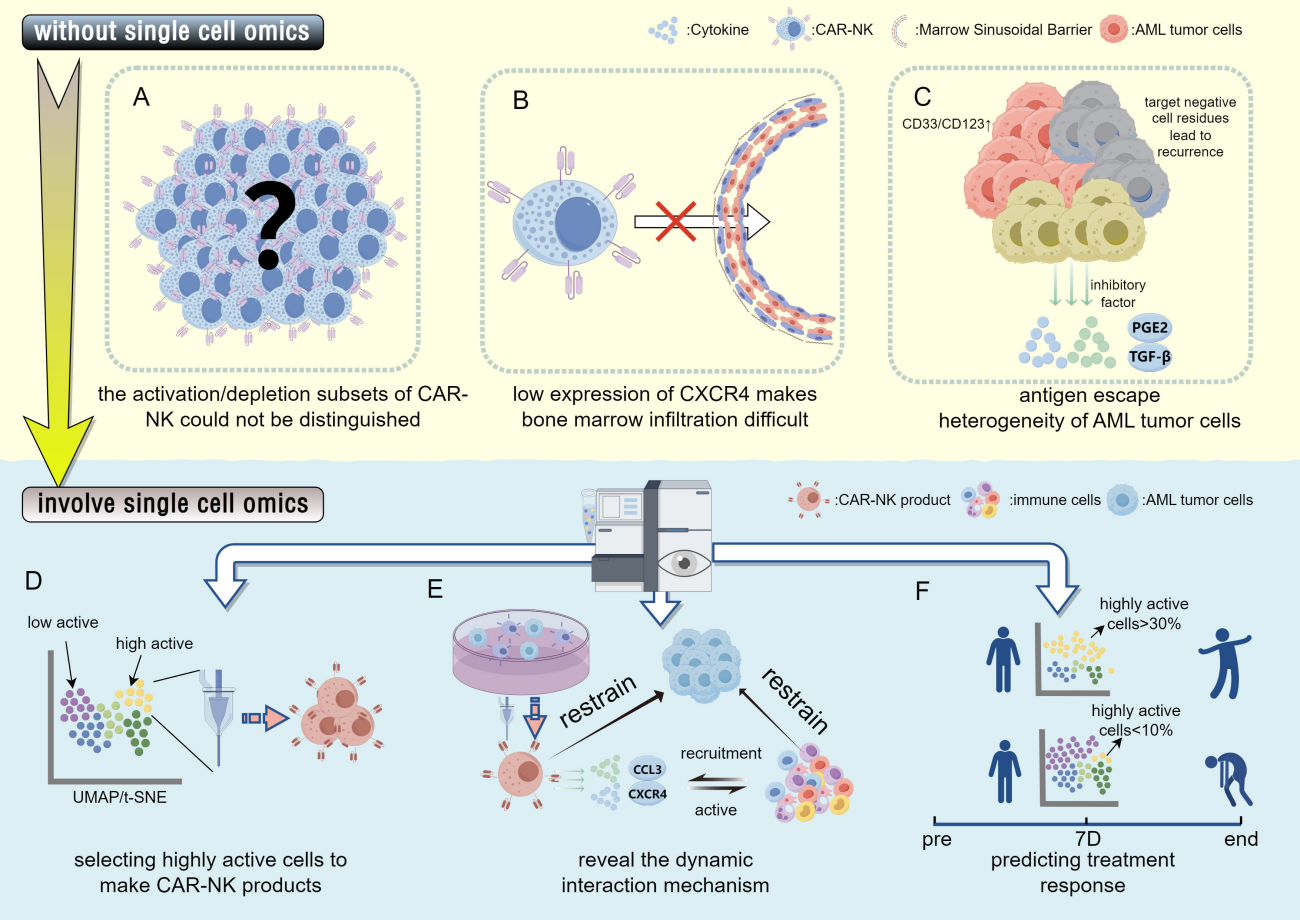
Single-cell omics-driven optimization strategies for CAR-NK therapy in AML. **(A-C)** Limitations of conventional CAR-NK therapy in the absence of single-cell omics insights. **(A)**Functional “black box”: A homogeneous population of CAR-NK cells obscures functional heterogeneity. The inability to distinguish activated from exhausted subpopulations leads to inconsistent product potency. **(B)** Inadequate BM infiltration: CAR-NK cells with low CXCR4 expression are impeded by the BM sinusoid barrier, preventing effective homing to and accumulation within the leukemic niche. **(C)** Antigen escape: The heterogeneous AML tumor microenvironment contains subclones with low or negative expression of target antigens, which evade recognition and eradication, ultimately leading to disease relapse. **(D-F)** Single-cell omics enables precise optimization of CAR-NK therapy through three key advances: **(D)** Resolution of functional subsets: scRNA-seq analysis (t-SNE/UMAP plot) resolves CAR-NK cells into distinct clusters, identifying a highly active subpopulation and an exhausted subpopulation. This enables fluorescence-activated cell sorting (FACS) to enrich for the highly active subset for product manufacturing. **(E)** Revelation of dynamic interaction mechanisms: upon co-culture with AML cells, an activated CAR-NK subpopulation secretes chemokines (CCL3, CCL4), recruiting and activating broader immune responses. This insight rationalizes combination strategies, such as CAR-NK with NKG2A blockade, achieving a 60% reduction in tumor burden. **(F)** Prediction of therapeutic response: Longitudinal monitoring via scRNA-seq allows for early prediction of clinical outcome. Patients with >30% “activated” CAR-NK cells at Day 7 post-infusion achieve complete remission (CR), whereas those with <10% activated cells show no response, enabling early intervention and personalized therapy.

Despite the inherent advantages of CAR-NK cells, several critical limitations—primarily antigen escape, functional heterogeneity, and suboptimal homing—continue to hinder their clinical efficacy in AML. Single-cell omics is currently transforming these challenges into actionable engineering strategies by resolving the complex landscape of clonal and functional diversity. The issue of tumor heterogeneity is a major driver of antigen escape; for instance, approximately 20–30% of AML patients harbor *CD33*-negative subclones, and the highly variable expression of *CD123* often leads to inconsistent responses in single-target therapies (92)(**Figure 4C**). To circumvent this, single-cell multi-omics has been employed to map the combinatorial expression landscape of surface antigens (such as *CD33*, *CD123*, and *CLL-1*) specifically on leukemic stem cells. This high-resolution, data-driven approach allows for the identification of optimal dual-target combinations, facilitating the design of tandem or logic-gated CARs that minimize the risk of relapse driven by clonal evolution (135).

Beyond antigen recognition, scRNA-seq has been pivotal in resolving the intrinsic functional “black box” that once obscured CAR-NK product potency (**Figure 4A**). While 10–15% of cells within a product typically express high levels of exhaustion markers (e.g., *TIGIT*, *NKG2A*) and exhibit low metabolic activity (124, 133), single-cell profiling has successfully resolved these cells into distinct clusters (**Figure 4D**). By distinguishing a “highly active” subpopulation (marked by *PCNA*, *PRF1*, and *CCL3*) from an “exhausted” subset (high *S100A8/A9*), single-cell insights provide precise molecular markers for fluorescence-activated cell sorting (FACS) to enrich high-potency effectors. Furthermore, the discovery of an expanded *CCL3/4*high activated CAR33-NK subpopulation post-culture has revealed dynamic interaction mechanisms where these cells recruit and activate broader immune responses (**Figure 4E**).This provides the mechanistic rationale for combining CAR-NK therapy with *NKG2A* blockade, a strategy that achieved a 60% tumor reduction in preclinical models (124, 132).

Finally, single-cell transcriptomics addresses the persistent challenge of inadequate BM infiltration caused by the sinusoid barrier (**Figure 4B**).Given that only 15–20% of iPSC-derived CAR-NK cells natively express *CXCR4*, their ability to penetrate the *CXCL12*-rich BM niche is often limited (134). By highlighting the specific downregulation of *CXCR4* and *SELPLG* during ex vivo expansion, scRNA-seq guided the genetic overexpression of *CXCR4* to restore chemotaxis and improve leukemia clearance. These longitudinal single-cell analyses further enable the transformation of clinical monitoring into dynamic tracking for the prediction of therapeutic response (**Figure 4F**).Clinical data indicate that a high percentage of “activated” CAR-NK cells (high *IFNG*, *TNF*) on day 7 correlates with a 75% complete remission rate, whereas low percentages indicate a lack of response (40, 123). Collectively, these insights underscore the clinical value of monitoring time-dependent phenotypic evolution to optimize product quality and facilitate personalized intervention.

#### 4.2.3 Comparative assessment and the complementary potential of multi-omics

As previously mentioned, scRNA-seq is the gold standard for subset identification; however, its “transcriptomic snapshots” often fail to predict long-term persistence. As evidenced by Merino et al. and Khan et al. (136, 137), scATAC-seq is superior for trajectory analysis and fate prediction, as it detects “epigenetic pre-programming” (e.g., chromatin closure at PRDM1 or TOX) that significantly precedes transcriptomic decline. Yet, epigenetic and transcriptomic landscapes often decouple from functional reality due to post-transcriptional regulation. For CAR-NK characterization and target selection, Perna et al. demonstrated that high-throughput proteomics is essential to bypass “pseudo-targets” identified by RNA-seq (135). By integrating proteomics and transcriptomics, they identified optimal dual-target combinations (e.g., CD33, CD123) that minimize off-tumor toxicity—a solution the transcriptome alone cannot provide. Despite protein-level accuracy, suspension-based omics lose spatial context, masking “immune-cold” regions where CAR-NK cells are physically excluded. ST is thus required to map the BM architecture (138), guiding the engineering of homing receptors (e.g., CXCR4) to overcome infiltration barriers. Finally, single-cell proteogenomics links these phenotypic shifts back to DNA mutations, resolving the clonal evolution that drives “antigen-low” relapse (41). This hierarchical framework transforms CAR-NK development from descriptive observation to predictive, spatially-aware engineering.

The convergence of these high-dimensional datasets into integrated multi-omic frameworks, particularly when empowered by AI, offers a transformative path from descriptive observation to predictive engineering in AML precision medicine. By bridging the gap between genotype and phenotype through CITE-seq or multi-modal assays, researchers can now identify molecular benchmarks for durable remission—such as the early epigenetic stability of the *IFNG* locus—which have proven superior to conventional flow cytometry in prognostic accuracy (139). Even more promising is the application of deep-learning architectures like total VI (140), which facilitate in silico perturbation experiments to model how dual-targeting CAR-NK constructs (e.g., *CD33/CLL-1*) withstand the metabolic and oxidative stressors of the leukemic marrow. Looking ahead, the profound fusion of multi-omics with spatial cartography is poised to underpin the development of “epigenetically armored” CAR-NK products. These next-generation therapies will be characterized not only by high-precision homing but also by a programmed functional resilience, maintained via epigenetic reprogramming, to ensure sustained efficacy within the hostile AML microenvironment—shifting clinical outcomes from transient responses to durable cures.

## 5 Future outlook

The therapeutic landscape for AML is undergoing a pivotal transition from empirical regimens toward a paradigm of dynamic surveillance and precision engineering. This evolution is predicated on resolving the molecular “identity crisis” of NK cell impairment, a task that demands the synthesis of disparate data layers into a unified, predictive framework. However, constructing this framework is hindered by critical knowledge gaps. Most notably, a systematic, single-cell comparison of NK cells between the BM and PB within the same AML patient—especially across multiple disease time points—remains a significant research gap. While the overarching dysfunctional trends are similar, the leukemic BM niche likely drives more severe and qualitative alterations in NK cell states, including a greater accumulation of immature phenotypes and intensified local suppression, compared to the PB compartment. How AML dynamically remodels the distinct baseline NK cell landscapes of BM and PB over time is unclear. Current single-cell studies have deeply characterized NK cell dysregulation within the leukemic BM niche, but a longitudinal, paired multi-omics analysis across compartments is lacking. Addressing this gap is biologically and clinically imperative: it will disentangle systemic immune dysfunction from niche-specific suppression, map the dynamic trajectories of NK cell exhaustion, and identify trafficking signatures—ultimately providing powerful biomarkers and revealing precise therapeutic targets.

Closing this gap requires transcending the inherent heterogeneity of AML through systematic data harmonization. Small-cohort studies catalog markers of dysfunction but struggle to distinguish patient-specific stochasticity from conserved mechanisms of immune evasion. Future progress hinges on aggregating high-dimensional, multi-compartment (BM/PB) datasets across genetic subtypes. AI-augmented meta-analyses can then distill universal molecular benchmarks of NK cell failure—whether conserved epigenetic “scars” or decoupled proteomic patterns—into a standardized lexicon. This will enable the categorization of patients into distinct immune-response trajectories at diagnosis and facilitate the detection of “nicle-specific” exhaustion as an early warning signal of relapse.

Yet, molecular signatures are context-dependent. Their full meaning emerges only from their spatiotemporal choreography within the BM niche. Critically, applying spatial omics to “liquid” malignancies like AML faces distinct hurdles, including ill-defined tissue architecture and sampling bias from limited tissue regions. However, recent advances—such as high-definition whole-slide platforms (e.g., Visium HD), optimized protocols for BM biopsy processing, and computational imputation methods guided by single-cell references—are beginning to overcome these limitations. The future integration of longitudinal, multi-time-point profiling with these advanced spatial technologies and 3D tissue-clearing will finally illuminate the “NK-leukemia” interactome *in situ*. This four-dimensional cartography is essential to delineate the boundary between reversible suppression and terminal exhaustion, transforming static snapshots into a real-time molecular dashboard for clinical decision-making.

These spatiotemporal insights form the fundamental blueprint for engineering next-generation NK cell products. While CAR-NK therapy addresses antigen-low escape, it marks only the inception. The next frontier involves engineering functional resilience: utilizing CRISPR-mediated epigenetic editing to fortify NK cells against the metabolic hostility of the AML niche. Furthermore, spatial data will guide the engineering of optimized homing receptors (e.g., enhanced CXCR4/CXCL12 axes) to penetrate immune-cold sanctuaries where leukemic stem cells hide.

The clinical translation of these high-dimensional designs depends on closing the validation loop. Sophisticated patient-derived xenograft models and BM organ-on-a-chip systems that recapitulate the longitudinal, multi-compartment evolution of the human niche are crucial. By validating computational predictions through high-throughput perturbational assays, the field can shift from cataloging impairment to actively correcting it.

This integrated cycle—iterating between big-data harmonization across compartments and time, spatiotemporal tracking, and the delivery of “epigenetically armored” cell products—promises to redefine the AML treatment arc. By finally defining the compartment-specific and time-resolved states of NK cells, we can move beyond transient remissions toward the goal of durable, lifelong cures.
